# Adenosine A_2A_Receptors in Substance Use Disorders: A Focus on Cocaine

**DOI:** 10.3390/cells9061372

**Published:** 2020-06-01

**Authors:** Karolina Wydra, Dawid Gawliński, Kinga Gawlińska, Małgorzata Frankowska, Dasiel O. Borroto-Escuela, Kjell Fuxe, Małgorzata Filip

**Affiliations:** 1 Department of Drug Addiction Pharmacology, Maj Institute of Pharmacology Polish Academy of Sciences, 31-343 Kraków, Poland; wydra@if-pan.krakow.pl (K.W.); gawlin@if-pan.krakow.pl (D.G.); kingaw@if-pan.krakow.pl (K.G.); frankow@if-pan.krakow.pl (M.F.); 2 Department of Neuroscience, Karolinska Institutet, 171 77 Stockholm, Sweden; Dasiel.Borroto.Escuela@ki.se (D.O.B.-E.); kjell.fuxe@ki.se (K.F.); 3 Section of Physiology, Department of Biomolecular Science, University of Urbino, Campus Scientifico Enrico Mattei, Via Ca’ Le Suore 2, 61029 Urbino, Italy; 4 Observatorio Cubano de Neurociencias, Grupo Bohío-Estudio, Zayas 50, Yaguajay 62100, Cuba

**Keywords:** adenosine (A)2 receptors, A_2A_-D_2_ receptor interaction, behavioral effects, drugs of abuse, laboratory animals, neurochemistry, striatum, substance use disorder

## Abstract

Several psychoactive drugs can evoke substance use disorders (SUD) in humans and animals, and these include psychostimulants, opioids, cannabinoids (CB), nicotine, and alcohol. The etiology, mechanistic processes, and the therapeutic options to deal with SUD are not well understood. The common feature of all abused drugs is that they increase dopamine (DA) neurotransmission within the mesocorticolimbic circuitry of the brain followed by the activation of DA receptors. D_2_ receptors were proposed as important molecular targets for SUD. The findings showed that D_2_ receptors formed heteromeric complexes with other GPCRs, which forced the addiction research area in new directions. In this review, we updated the view on the brain D_2_ receptor complexes with adenosine (A)2A receptors (A_2A_R) and discussed the role of A_2A_R in different aspects of addiction phenotypes in laboratory animal procedures that permit the highly complex syndrome of human drug addiction. We presented the current knowledge on the neurochemical in vivo and ex vivo mechanisms related to cocaine use disorder (CUD) and discussed future research directions for A_2A_R heteromeric complexes in SUD.

## 1. Introduction

From the perspective of public health, substance use disorders (SUD; commonly called drug addictions) are unsolved issues [1]. Several drugs can lead to addictive behavior in humans and animals,and these include psychostimulants, opioids, cannabinoids (CB), nicotine, and alcohol. The etiology and mechanistic processes,as well as the therapeutic options to deal with SUD, are neithercomplete nor well-understood (e.g., [2]). Thus, researchers must search for the neurobiological bases of development to uncover efficient treatments for this disorder, which affects the global population. SUD is a chronic brain disorder, in which, after the initial behavioral spectrum of feeling (well-being, pleasure, and euphoria), compulsive drug-seeking and drug-taking behaviors appear despite the negative consequences, and relapses, accompanied by psychic, somatic, and vegetative disturbances, are triggered after drug abstinence [3]. These behavioral outcomes are realized through distinct effector mechanisms, including neurotransmitter transporters, ion channels, and receptor proteins.

It is well-established that drugs of abuse have common features that trigger addictive potential, i.e., they increase dopamine (DA) neurotransmission within the mesocorticolimbic circuitry of the brain from the ventral tegmental area, the nucleus accumbens, and the prefrontal cortex. Increased DA neurotransmission leads to indirect activation of five types of DA receptors, among which, D_2_ receptors have been the most widely studied as molecular targets for SUD. The findingsshowed that D_2_ receptors formed heteromeric complexes with other G protein-coupled receptors(GPCRs), while such heteromersdemonstrated pharmacology and functions distinct from the constituent receptors,which forced the addiction research to explore new directions.

Here, we update the view on the brain D_2_ receptor complexes with adenosine (A)2A receptors (A_2A_R), the role of A_2A_R in different aspects of addiction phenotypes in laboratory animal procedures that permit the highly complex syndrome of human drug addiction (drug-induced reward, discrimination, sensitization, seeking behavior, and withdrawal symptoms). At the end, we present the current knowledge on the neurochemical in vivo and the ex vivo mechanisms related to cocaine use disorder (CUD) and discuss future research directions for A_2A_R heteromeric complexes in SUD.

## 2. A_2A_Rs and Their Localization in the Brain

The distribution of A_2A_Rs in the mammalian brain on the level of transcript and protein was demonstrated ex vivo using reverse transcriptase-polymerase chain reaction (RT-PCR), Northern blotting, Western blotting, ELISA, in situ hybridization, immunohistochemistry, autoradiography, and radioligand binding, while position emission tomography (PET) studies were employed for the in vivo analyses.

Several studies indicated the differential expression of A_2A_Rs in mammalian brains. Studies performed with techniques to detect mRNA encodingA_2A_Rsdemonstrated, in rodents, the highest receptor transcript level in the striatal areas, while lower levelswere detected extrastriatally in the hippocampus, hypothalamus, thalamus, cerebral cortex, amygdala, thalamic nuclei, and the substantia nigra [4,5,6,7,8,9,10,11]. A similar pattern of A_2A_R mRNA expression was observed in the human brain with high levels in the nucleus caudatus, while much lower levels were found in the optical cortex, amygdala, hippocampus, substantia nigra, and the cerebellum [12]. The highest protein density was reported for the striatum (dorsal and ventral parts), olfactory tubercle, and globus pallidus in the mammalian brain [8,9,13,14,15].

**Figure 1:**
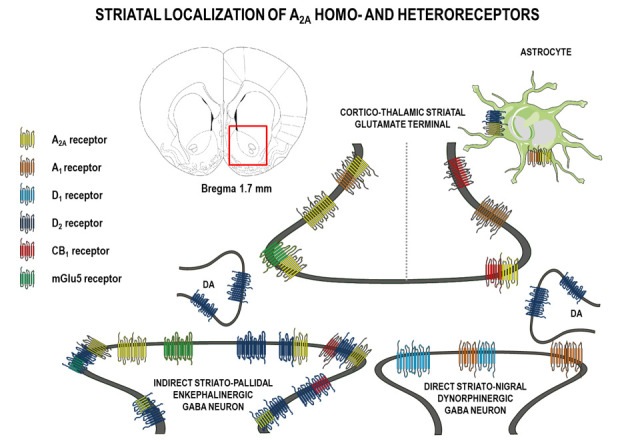
Schematic diagram showing the neuronal and astrocytic localization of adenosine (A)2A homo-, heterodimer, and oligomer complexes with dopamine (D), cannabinoid (CB), and metabotropic glutamate receptors (mGlu) on glutamatergic input from the cortex and thalamus and dopaminergic (DA) input from the ventral tegmental area and both enkephalinstriato–pallidal and dynorfinstriato-nigral gamma-aminobutyric acid (GABA) neurons in the rat striatum. To simplify, cholinergic interneurons are not included.

Using anatomical, electrophysiological, and biochemical approaches researchers demonstrated that A_2A_R in brainswerelocalized on neurons and glia cells (including astrocytes and microglia) [7,8,9,16,17]. On neurons, these receptors occurredpostsynaptically (on dendrites and dendritic spines), presynaptically (on terminals of axon collaterals), and extrasynaptically (on somas) [7,8,9,18,19] (Figure 1).

In the striatum, A_2A_Rs were localized on neurons (ca. 90%) and about 3% were on astrocytes [9,20,21,22,23]. On striatal neurons, about 70% of A_2A_Rs were located postsynaptically, 23% presynaptically, and 3% extrasynaptically [9,21].

Postsynaptically-expressed striatal A_2A_Rs were mainly localized to the gamma-aminobutric acid (GABA)ergic medium-sized spiny neurons (MSN) of the indirect pathway projecting to the external segmentofthe globus pallidus. These latter neurons also expressed a high density of DA D_2_ receptors and enkephalin [7,9,10,13,14,24,25]. Inversely, neurons of the direct striato-nigral pathway (which selectively expressed DA D_1_ receptors and the peptide dynorphin) did not contain a significant level of A_2A_Rs [13]. Postsynaptically localized A_2A_Rs were found around dopaminergic synapses [21]. In the striatum, a smaller amount of A_2A_Rs were localized presynaptically mainly on cortico-thalamic glutaminergic terminals that contacted medium-sized spiny neurons of the GABAergic direct and indirect pathways [9,19,26]. Striatal A_2A_Rs were located presynaptically on cholinergic neurons that modulated acetylcholine release [27,28,29].

Using double immunofluorescence, co-immunoprecipitation, resonance energy transfer techniques (a sequential resonance energy transfer (SRET) and bimolecular fluorescence complementation plus bioluminescence response energy transfer (BRET)), and in vivo proximity ligation assay (PLA), research indicated that A_2A_Rs occur in dimers:They form either homodimers (A_2A_R-A_2A_R) or heterodimers with other metabotropic GPCRs.

In the hippocampus,the following occurred:

- A_1_-A_2A_, A_2A_-A_2B_, A_2A_-A_3_isoreceptor complexes were found in moderate to high densities in the dorsal hippocampus, mainly involving the pyramidal cell layer using in situ PLA [30,31,32,33].

In the striatum, the following existed:

- homodimeric A_2A_R-A_2A_R complexes on the cell surface, as shown using BRETassays [31];
- A_2A_R heterodimers that interacted with other receptors, such as A_1_, A_2B_, A_3_, CB_1_, D_2_, D_3_, glutamatergic Glu type 5 (mGlu5), fibroblast growth factor receptor FGFR1), and Sigma1 receptors [18,30,31,32,33,34,35].

A_1_-A_2A_isoreceptor complexes were identified in striatal glutamate nerve terminals at the presynaptic level [36]. On striatal glutamate nerve terminals, there may also exist A_2A_-D_2_ heterodimers in balance with A_1_-A_2A_-D_2_ trimeric heteroreceptor complexes.

In the striatum in vivo and in vitro A_2A_Rs and mGlu5 receptor complexes were detected inter alia extrasynaptically on glutamatergic terminals and GABA dendrites, where they play a role in local plasticity and in modulation of the activity of GABA striato–pallidal neurons [26,37]. Activation of A_2A_Rs and mGlu5 receptors resulted in decreased affinity of D_2_ for DA [38], increased striatal c-Fos expression [37] and cyclic adenosine monophosphate (cAMP) formation, and striatal DA- and cAMP-regulated neuronal phosphoprotein DARP-32 phosphorylation [39]. In addition, presynaptic interactions between A_2A_Rs and mGlu5 receptors on cortico–striatal glutamatergic nerveterminals may also contribute to the described interaction by synergistic regulation of glutamate release [26].

In the striatum, A_2A_Rs and cannabinoid CB_1_ receptors may also form heteromeric complexes and, in this way, A_2A_R activation facilitated CB_1_ receptor signaling [40,41]. There is also evidence for A_2A_-CB_1_-D_2_ and A_2A_-D_2_-mGlu5 receptor heteromers in transfected cells [42,43] and such interactions appeared at both pre- and postsynaptic levels to control the neurotransmission and signaling in different brain structures. The existence of A_2A_-CB_1_-D_2_ receptor mosaics was found in the terminal parts of the dorsal striato–pallidal GABA neurons, in the dendritic regions of the ventral striato–pallidal GABA neurons, and in the cortico–striatal glutamate terminals [42,44,45,46]. The A_2A_R-mGlu5 receptor complexes existedinter alia on the glutamate terminals, forming synapses on the striato–pallidal GABA neurons [26,36,47].

Flajolet, Greengard, and colleagues discovered FGFR1and A_2A_R complexes [48,49] as well as FGFR1-A_2A_-D_2_ heterocomplexes in the striatum [50]. In the FGFR1-A_2A_-D_2_heterocomplex, the adenosine-induced activation of A_2A_R may then enhance signaling over FGFR1 via an allosteric receptor–receptor interaction, increasing the structural plasticity and repair.

A_2A_Rs can heteromerize also with D_2_, D_3_, and D_4_ receptors [34,51,52]. The D_3_ receptor in the brain is mainly found in the ventral striatum, unlike the D_2_ receptor, which is located in high densities in both the ventral and dorsal striatum. Rivera et al. demonstrated that the D_4_ receptor was enriched in the striosomes and the matrix of the dorsal striatum [53]. Borroto-Escuela et al. demonstrated A_2A_-D_4_ heteroreceptor complexes in the dorsal striatum, especially in the striosomes [34] using an in situ PLA assay [33].

- The first recognized and best known heteromeric interaction was found between the A_2A_Rs and D_2_ receptors, by which the activation of A_2A_ (G_s/olf_-coupled) receptors interfered with coupling of D_2_ receptors to G_i_ isoforms of G proteins [54,55,56]. A_2A_-D_2_ heteroreceptor complexes with antagonistic receptor–receptor interaction were found to exist on:
- The dorsal and ventral striato–pallidal GABA (antireward) pathway [44,51];
- the cortico–striatal glutamate nerve terminals, where the A_2A_R protomer inhibited the D_2_ receptor protomer-induced inhibition of glutamate release [47];
- striatal cholinergic interneurons [57];and
- striatal astrocytes [58,59].

The striatal A_2A_R-D_2_ receptor heterocomplexes were first shown by Trifilieff et al. [60] using anin situ PLA assay and, later, by Borroto-Escuela and his group [61].

Based on a mathematical and bioinformatic approach,Tarakanov and Fuxededuced a set of triplet homologies (triplet puzzle) contributing to the formation of the receptor interface [62]. Using thein vivo PLA assay technique,theydemonstrated a high expression of A_2A_-D_2_-Sigma1 heterocomplexes was indicated to exist in the ventral striatum [60,61,63].

## 3. A_2A_R and Behavioral Actions to Cocaine and Other Drugs of Abuse—In Vivo Evidence

Numerous preclinical studies confirmed functional interactions between A_2A_Rs and drugs of abuse with different mechanisms of action on the central nervous system. Using classical pharmacological tools (agonists or antagonists of A_2A_Rs), as well as animal genetic models (overexpression or knockout (KO) of A_2A_Rs) studies demonstrated an involvement of these receptors in the locomotor response, drug discrimination, reward-seeking behavior, and withdrawal syndrome associated with the use of psychostimulants (cocaine, amphetamine, methamphetamine, and 3,4-methylenedioxymethamphetamine (MDMA)), nicotine, opioids (morphine and heroin), cannabinoids (∆9-tetrahydrocannabinol (THC)), and ethanol.

### 3.1. Locomotor Responses to Drugs of Abuse

Several studies indicated a role of A_2A_Rs in the locomotor action of drugs of abuse. Thus, studies using A_2A_R agonists (2-[(2-aminoethylamino)carbonylethylphenylethylamino]-5′-*N*-ethylcarboxamidoadenosine (APEC) and (4-[2-[[6-Amino-9-(*N*-ethyl-b-d-ribofuranuronamidosyl)-9H-purin-2-yl]amino]ethyl]benzene-propanoic acid hydrochloride (CGS 21680)) consistently indicated the inhibitory effect of activation of these receptors in psychostimulant-induced locomotor hyperactivity. The administration of APEC (0.01 mg/kg) [64] or CGS 21680 (0.1–2.0 mg/kg) [65] decreased locomotor response to acute amphetamine treatment, while CGS 21680 (0.03–3.0 mg/kg) attenuated such behavior of methamphetamine in rats [65,66]. In non-habituated mice, CGS 21680 (0.01–0.5 mg/kg) inhibited both amphetamine- and cocaine-induced hyperlocomotion; however, in the case of amphetamine, this agonist was effective only the highest used dose [67]. Locomotor hyperactivation induced by acute cocaine administration was decreased by CGS 21680 (0.2 mg/kg) also in well-habituated rats [68]. Supporting the pharmacological studies, A_2A_R overexpressing rats—as opposed to wild-type controls—did not induce an increase in locomotion after a single cocaine injection [69].

In contrast to the stimulation of A_2A_Rs, pharmacological antagonism enhanced psychostimulant-induced hyperlocomotion. The A_2A_R antagonists 3,7-dimethyl-1-propar-gylxanthine (DMPX; 3.0–6.0 mg/kg) [70] and (3-(3-hydroxypropyl)-8-(m-methoxystyryl)-7-methyl-1-propargylxanthine phosphate disodium salt (MSX-3; 5.0–25.0 mg/kg) [68] increased the locomotor hyperactivation after acute cocaine or amphetamine administration, respectively. In a recent study, Haynes et al. [71] used presynaptic 2-(2-furanyl)-7-[3-(4-methoxyphenyl)propyl]-7H-pyrazolo[4,3-e-][1,2,4]triazolo[1,5-c]pyrimidin-5-amine (SCH 442416; 1.0 mg/kg) and postsynaptic 8-[(E)-2-(3,4-dimethoxyphenyl)etheyl]-1,3-diethyl-7-methylpurine-2,6-dione (KW 6002; 1.0 mg/kg) A_2A_R antagonists and showed that only a postsynaptic blockade of these receptors increased the locomotor activity induced by acute cocaine treatment in habituated rats.

Animal genetic models did not explicitly confirm the above pharmacological observations. Thus, Chen et al. [72] demonstrated that locomotor responses to amphetamine and cocaine were attenuated in mice lacking A_2A_Rs, while Wright et al. [73] did not observe changes in locomotion after cocaine administration in A_2A_R KO animals while hyperactivity following chronic methamphetamine was attenuated.

To explain the differences between pharmacological inhibition and genetic elimination, as well as better understanding of the role A_2A_Rs in the modulation of psychostimulant hyperactivity, a study was conducted in which cocaine-induced hyperactivity was enhanced in striatum-specific A_2A_R KO mice (selective deletions of A_2A_Rs only in the neurons of the striatum) and attenuated in forebrain-specific A_2A_R KO mice (deletions A_2A_Rs in the neurons of striatum, cerebral cortex, and hippocampus). In addition, the administration of a selective A_2A_R antagonist—KW 6002 (3.3 mg/kg)—into striatum-specific A_2A_R KO mice attenuated the cocaine effects, in contrast to the enhanced cocaine effects observed in wild-type mice. Therefore, these results highlight the opposing modulation in locomotor responses to cocaine—the stimulatory role of A_2A_Rs in the extrastriatalneurons and the inhibitory action of A_2A_Rs in the striatal neurons [74].

Research has demonstrated that A_2A_R activity affects rodent behavioral responses not only after acute psychostimulant administration but also with chronic exposure. CGS 21680 (0.2 mg/kg), given during the development of cocaine sensitization, reduced the locomotor response to cocaine challenge dose following 5-day withdrawal. The inhibition of hyperlocomotion augmentation in rats after acute CGS 21680 (0.2 mg/kg) administration before a cocaine challenge was also observed [68]. In A_2A_R overexpressing animals, a challenge with cocaine evoked a slight increase in locomotor activity in comparison to wild-type rats [69]. Taken together, the stimulation/increased activity of A_2A_Rs protected against the development and expression of cocaine sensitization. Shimazoe et al. reported, that, in addition to affecting the effects of cocaine CGS 21680 at higher doses (1.0–3.0 mg/kg), this effect reduced the development, and in the lower dose (0.1 mg/kg), the expression of methamphetamine sensitization [75].

In addition to observations related to A_2A_Rs antagonism on acute psychostimulant injection, researchers demonstrated that MSX-3, in contrast to agonist (CGS 21680), enhanced the development and expression of cocaine sensitization [68]. On the other hand, in the case of cocaine, similar to the effects of single administration, studies showed that the behavioral effect (motor activity) differed depending on the ligand used. Hence, a postsynaptic (KW 6002; 1.0 mg/kg) A_2A_R antagonist had no effect on the expression of locomotor sensitization, while a presynaptic blockade with SCH 442416 (1.0 mg/kg) inhibited the expression of locomotor sensitization [71]. Another study, in turn, showed that in mice, a blockade of A_2A_Rs with 2-(2-furanyl)-7-(2-phenylethyl)-7H-pyrazolo[4,3-e][1,2,4]triazolo[1,5-c]pyrimidin-5-amine (SCH 58261; 0.03 mg/kg) or KW 6002 (0.03 mg/kg) prevented or delayed the development of sensitization to amphetamine [76].

Similar behavioral effects (limitation development of amphetamine sensitization) were observed in mice with a global lack of A_2A_Rs [77], in forebrain-specific A_2A_R conditional KO mice [76]. The enhanced motor response characteristic for the development of cocaine sensitization did not change in mice with the genetic inactivation of A_2A_Rs [78]. The literature providedlittle and inconsistent evidence regarding the influences of A_2A_Rs on locomotor responses to other drugs of abuse. It has only been proven that, in the case of opioids, CGS 21680 (0.1 mg/kg) significantly reduced the development of morphine hypersensitivity (with no increase in locomotor activity by the challenge morphine dose following the 7-day withdrawal) in mice induced by increasing doses or sporadic administration of this substance [79,80].

Castañé et al. [81] did not observe differences between mice lacking A_2A_Rs and wild-type littermates in acute effects induced by morphine (similar increase in locomotor activity after morphine injection was observed) [81]. In contrast to psychostimulants and morphine, THC administration decreased locomotor activity. In a study comparing the effect of THC (5–20 mg/kg) acute treatment in A_2A_R KO and wild-type mice resulted in similar hyperlocomotion in both experimental groups [82]. In a pharmacological study, an antagonist of A_2A_R MSX-3 (3.0 mg/kg) blocked the locomotor depressant responses observed after intrastriatal cannabinoid CB_1_ receptor agonist (WIN 55212-2) treatment [40]. The same antagonist (MSX-3; 2.0–4.0 mg/kg) increased locomotion during ethanol consumption in the Drinking-in-the-Dark paradigm in mice [83].

One study assessed the impact of deletions of A_2A_R on the locomotor effects of acute alcohol administration; however, depending on the genetic background, increases in hyperactivity were noted in mice (mice generated on CD1 background) or no changes were noted (mice generated on C57BL/6J background). In addition, there was no effect of a lack of A_2A_Rs in CD1 mice locomotor sensitization development induced by chronic ethanol administration [84]. In the use of nicotine, a pharmacological study showed that the A_2A_R agonist CGS 21680 (0.2–0.4 mg/kg) inhibited, and the A_2A_R antagonist KW 6002 (0.5 mg/kg) enhanced the acute locomotor effect of nicotine in rats.

An A_2A_R agonist (0.4 mg/kg), but not antagonist, modulated the development of nicotine induced locomotor sensitization (decrease in the locomotor response to the nicotine challenge), while, during evaluation, the expression of nicotine sensitization, stimulation of A_2A_R was reduced, and a blockade of this receptor increased the locomotor response to a nicotine challenge [85]. In turn, acute nicotine treatment induced a reduction of horizontal movements in A_2A_R KO mice and wild-type littermates [86].

The above data provided strong evidence for the pre-/post-synaptic and brain structure-dependent involvement of A_2A_Rs in the locomotor response of animals to psychostimulants and morphine treatment. At the same time, more research is needed to clearly assess their involvement in the motor effects associated with the use of other substances.

### 3.2. Discrimination

The drug discrimination paradigm is a well-established behavioral procedure to model the subjective effects of drugs in animals. So far, the available research on A_2A_Rs-drugs of abuse interaction demonstrated that, in rats trained to discriminate methamphetamine or cocaine from its vehicle (saline) under a fixed ratio (FR)10 schedule of food presentation, A_2A_R agonist CGS 21680 (0.056–0.177 mg/kg) shifted the cocaine dose–response curve markedly to the left and produced partial, but significant cocaine-lever selection in substitution tests [87]. The same authors found that CGS 21680 produced neither substitution for the methamphetamine-training stimulus response nor a change in its dose–response curve in the combination protocol. For both psychostimulants, CGS 21680 reduced animal response rates in the doses used. A pharmacological blockade of A_2A_Rs with MSX-3 (3–56 mg/kg) significantly shifted the dose–response curves of cocaine and methamphetamine to the right as well as caused high levels of drug-lever selection for both psychostimulants in substitutions tests [87,88].

These observations were consistent with previous reports in which another A_2A_R antagonist, DMPX (5.6 mg/kg), in rats trained to recognize methamphetamine from saline shifted the drug dose–response curve to the left while in doses of 10–18 mg/kg, it led to generalization of the methamphetamine-training stimulus. Methamphetamine-like effects of this A_2A_R antagonist were blocked partially by the DA D_1_ receptor antagonist (R-(+)-7-chloro-8-hydroxy-3-methyl-1-phenyl-2,3,4,5-tetrahydro-1H-3-benzazepine hydrochloride (SCH-23390) and fully by the DA D_2_ receptor antagonist spiperone, which indicates the interaction between adenosine and dopaminergic signaling in the control of subjective effects of this drug of abuse [89].

### 3.3. Reward

In preclinical studies, the rewarding effects of drugs of abuse are evaluated in self-administration or conditioned place preference (CPP) paradigms. In 2001, the first report indicated that in rats trained for intravenous cocaine self-administration (0.6 mg/kg/infusion) under a FR5 schedule of reinforcement, the A_2A_R agonist CGS 21680 (0.1–0.4 mg/kg) reduced the number of drug infusions [90]. The reduced number of cocaine infusions was mainly due to the prolonged time of responding to the first drug infusion at the beginning of the session, and this effect was associated, among others, with the sedative effects of CGS 21680 [90]. The same agonist (0.2–0.4 mg/kg) produced a downward shift in the cocaine dose–response curve and,more importantly, CGS 21680 reduced the number of active lever presses and cocaine (0.5 mg/kg/infusion) reinforcements defined as the breaking point in the progressive ratio (PR) reinforcement schedule[91].

The latter authors found also that none of the CGS 21680 effective doses changed the inactive lever responses, which indicated the drug specificity. Wydra and colleagues [92] also pointed to the important role of the localization of A_2A_Rs in controlling the rewarding effects of cocaine. Namely, in the intra-accumbal shell, but not the intra-infralimbic prefrontal cortex, microinjections of CGS 21680 (1.0–2.5 ng/side) dose-dependently decreased cocaine (0.25 mg/kg/infusion) self-administration. Interestingly, the CGS 21680 (0.1 mg/kg)-induced reduction of the number of presses on the active lever and the number of cocaine infusions (0.25 mg/kg/infusion) was not changed by the A_2A_R synthetic trans membrane (TM) 2 peptide (0.1 µM/0.5 µL/min/side) bilaterally microinjected into the nucleus accumbens [93], while the same dose of a local administration of the TM 5 peptide (which disrupts A_2A_-D_2_ heteroreceptor complexes) counteracted the inhibitory effects of CGS 21680 on cocaine self-administration [94].

Supporting the self-administration paradigm, stimulation of A_2A_Rs with CGS 21680 (0.25–0.5 mg/kg) decreased the acquisition and expression of cocaine-induced CPP [70] or amphetamines [95], while the drug, in a dose of 0.03 mg/kg,also inhibited the development of methamphetamine-induced CPP [66]. However, a CGS 21680-induced reduction of drug reward was not observed, after stimulation of A_2A_Rs with this agonist in low doses (0.01–0.03 mg/kg) on methamphetamine self-administration (0.05–0.1 mg/kg/infusion) under FR5 or a progressive ratio schedule of reinforcement [66].

If pharmacological stimulation of A_2A_Rs attenuated the drug reward, the tonic activation of these receptors with selective antagonists seemednot to be linked to drug motivational or reinforcing effects. Thus, neither systemic administration of MSX-3 (3.0 mg/kg) nor systemic or local (into the nucleus accumbens or prefrontal cortex) microinjections of KW 6002 (1.0–2.5 μg/side) or SCH 58261 (1.0–2.5 µg/side) altered cocaine self-administration in squirrel monkeys [96] or rats [92].

In line with the above findings, A_2A_R KO animals showed no behavioral differences compared to the control mice in a cocaine–induced CPP paradigm[78]. Self-administration procedures provided evidence that, in A_2A_R KO mice, the cocaine reward was reduced compared to wild-type littermates under FR1 on FR3 schedules. The deletion of A_2A_Rs resulted in a reduction in the maximum effort to obtain a cocaine infusion under a PR schedule of reinforcement [78]. Such inhibition was also observed for MDMA self-administration under an FR1 schedule of reinforcement in A_2A_R KO mice [97].

The above data suggested that A_2A_Rs participate in the control of the rewarding and motivational properties of psychostimulants, the effects of CGS 21680 in animal paradigms to evaluate such actions are drugs of abuse-sensitivity, while the effects of blockade of these receptors by receptor specific ligands or via genetic modification are undefined so far.

Two studies evaluated the rewarding properties of nicotine. In the first, CGS 21680 (0.03–0.09 mg/kg) was effective in alleviating nicotine CPP, but only in male adolescent rats, suggesting a sex difference in adenosine signaling [98]. The second study showed that, in mice, the deletion of A_2A_Rs suppressed nicotine-induced CPP compared with wild-type animals [86].

The interaction between A_2A_Rs and opioids at the behavioral level showed that in rats CGS 21680 (0.01–0.05 mg/kg) increased the number of drug infusions during the acquisition of morphine self-administration, while the A_2A_R antagonist DMXP (0.25–1.0 mg/kg) reduced morphine self-administration. Interestingly, the same ligands, given repeatedly (before training sessions), had the opposite effect during the maintenance of morphine self-administration (CGS 21680 decreased, and DMXP increased the number of intravenous morphine injections) [99]. Using morphine-induced CPP, researchers demonstrated that neither CGS 21680 (0.025–0.05 mg/kg) nor SCH 58261 (0.5–1.0 mg/kg) significantly affected the expression of sensitization to the opioid in previously conditioned rats.

On the other hand, the same ligands inhibited the acquisition of sensitization to morphine-evoked CPP [100]. Supporting the last pharmacological study, the rewarding effects of morphine (5 or 10 mg/kg) in CPP were not completely observed in mice lacking A_2A_Rs as opposed to wild-type animals [81]. Confirming this observation, the deletion of A_2A_Rs significantly reduced morphine self-administration under the FR1 schedule and breaking point on the PR schedule, highlighting their participation in controlling the motivational properties of this opioid [101].

Other studies reported that a blockade of A_2A_Rs in squirrel monkeys with a low dose of its antagonist, MSX-3 (1 mg/kg), caused downward shifts of the THC and anandamide (the endogenous cannabinoid with CB_1_ and CB_2_ agonistic activity) dose–response curves [96]. The higher dose (3 mg/kg) of MSX-3 shifted the above CB dose–response curves to the left, testifying to their enhancement in the rewarding effects [96]. In addition, in A_2A_R KO mice, a reduction in the rewarding effects of THC was observed in the CPP model [82].

In studies on the rewarding effects of ethanol, CGS 21680 (0.5–1.0 mg/kg) reduced or did not affect the consumption and ethanol preferences in a two-bottle free-choice paradigm in mice [84]. In rats, a low dose of CGS 21680 (0.065 mg/kg) increased ethanol operant self-administration within non-dependent rats while higher CGS 21680 doses (0.095 or 0.125 mg/kg)—similar to the previous findings on mice—significantly reduced operant alcohol responses under FR1 within both non-dependent and dependent rats [102]. Recently, in rats overexpressing A_2A_Rs, no change in the ethanol drinking behavior during the acquisition/maintenance phase in the two-bottle choice paradigm was reported [103].

The findings with the selective A_2A_R blockade were also inconsistent. Thus, low doses of A_2A_R antagonist DMPX (1 mg/kg) increased, while higher doses (10–20 mg/kg) significantly reduced the operant ethanol self-administration in Long–Evans rats [104]. Similarly, in Wistar rats, DMPX (3–10 mg/kg) dose-dependently reduced ethanol operant reinforcement [105], while another A_2A_R blocker, SCH 58261 (2 mg/kg), reduced the response for ethanol in alcohol-preferring rats [106]. Using the “Drinking-in-the-Dark” paradigm in mice, researchers found that MSX-3 (1–4 mg/kg) did not change voluntary alcohol consumption [83], while 4-(2-[7-amino-2-(2-furyl)[1,2,4]triazolo[2,3-a][1,3,5]triazin-5-ylamino]ethyl)phenol (ZM241385; 20 mg/kg) increased the consumption and ethanol preference in a two-bottle free-choice paradigm in mice [107]. Further, the current literature with genetic animal models indicated that male and female A_2A_R KO mice generated on a CD1 background were characterized by increased ethanol drinking and alcohol preference compared with wild-type mice [108]. The same mice displayed a decrease ethanol-induced CPP, and, as in previous studies, consumed more ethanol with a higher preference, whereas, in the A_2A_R KO mice produced on a C57BL/6J background, no changes were observed [84].

Taken together, the pharmacological approach indicated that A_2A_Rs exerted inhibitory control over the reward properties evoked by drugs of abuse.

### 3.4. Seeking Behavior

In the context of substance use disorder therapy, relapse or drug-seeking behavior after a period of abstinence is the most serious limitation of effective treatment. In animal models, drug-seeking behavior is assessed by re-exposing animals to a previously used drug of abuse, the drug-associated context (cue) or stressor [109].

Many literature reports provided strong conclusions on the inhibitory effects of A_2A_Rs on cocaine-seeking behavior. Namely, the A_2A_R agonist CGS 21680 dose-dependently (0.03–0.3 mg/kg) inhibited cocaine-induced reinstatement (15 mg/kg) after a 7-day period of drug abstinence in Sprague–Dawley rats. At the same time, this agonist (0.03 mg/kg) also blunted cocaine-seeking behavior induced by quinpirole (a D_2_ dopaminergic receptor agonist) or a drug-associated cue [110].

Wydra et al. confirmed these observations in Wistar rats, in which CGS 21680 in a low dose range (0.05–0.4 mg/kg) significantly attenuated cocaine- (10 mg/kg;i.p.), quinpirole- and cue-induced reinstatement of cocaine-seeking behavior [111]. This effect may be associated with adenosine signaling in the rat nucleus accumbens, as reduced cocaine-induced (15 mg/kg), quinpirole-induced [112], or the cue plus the subthreshold dose of cocaine-induced (2.5 mg/kg) [92] reinstatement of cocaine seeking was reported following the intra-accumbal microinjection of CGS 21680 (2.5 ng/side), but not after pharmacological stimulation of the A_2A_Rs within the prefrontal cortex [92]. The effects of the A_2A_R stimulation with CGS 21680 (0.3 mg/kg) in methamphetamine-seeking behavior were partially consistent with the above observations for cocaine. Namely, this agonist blunted quinpirole-induced drug-paired lever responses, but did not affect methamphetamine-seeking induced by a methamphetamine priming injection in rats [113].

Contrary to the stimulation of A_2A_Rs, their accumbal and systemic blockade with MSX-3 (10 µg/side and 6 mg/kg, respectively) or SCH 58261 (5 µg/side) produced cocaine-seeking behavior [92,112]. In addition, intra-accumbal microinjection with MSX-3 (10 µg/side) [112], but not KW 6002 (2.5–5.0 µg/side) and SCH 58261 (2.5 µg/side) [92], potentiated the reinstatement of cocaine-seeking behavior induced only by sub-threshold doses of cocaine and quinpirole, suggesting that removing the tonic activity of A_2A_R enabled behaviors mediated by DA receptors [112]. Similarly, a systemic blockade of A_2A_Rs by KW 6002 (0.25–0.5 mg/kg) or SCH 58261 (2 mg/kg) [91], or KW 6002 (1–3 mg/kg) [71] in rats extinguished from cocaine self-administration or 9-chloro-2-(2-furanyl)-[1,2,4]triazolo[1,5-c]quinazolin-5-amine (CGS 15943; 0.032–0.32 mg/kg) in baboons [114] reinstated cocaine-seeking, and this effect was eliminated by the D_2_-like receptor antagonist raclopride (0.1–0.4 mg/kg) [111].

In contrast, KW 6002 at low doses (0.0625 mg/kg or 0.125 mg/kg), but not SCH 58261, evoked a reinstatement of cocaine-seeking behavior induced by sub-threshold dose of cocaine (2.5 mg/kg) or the drug-associated cue, respectively [111]. However, repeated blocking of presynaptic, but not postsynaptic, A_2A_Rs during extinction training with SCH 442416 (0.3 or 1 mg/kg) produced enduring reductions on cocaine- and quinpirole-induced reinstatement of cocaine-seeking behavior [115]. This finding was also confirmed by recent studies, in which KW 6002 (the postsynaptic A_2A_R antagonist), at a dose of 0.3 mg/kg, enhanced, while SCH 442416 (the presynaptic A_2A_R antagonist), at a dose of 1 mg/kg, reduced cocaine-induced reinstatement of cocaine seeking [71]. The above data, in addition to confirming the important role of A_2A_Rs to control cocaine-seeking behavior, indicated the antagonistic effect of A_2A_Rs and D_2_ receptors in cocaine relapse.

Few papers focused on assessing the role of A_2A_Rs in the seeking behavior of other drugs of abuse. Thus, Yao et al. [116] reported that, in rats, a pharmacological blockade of A_2A_Rs with systemic MSX-3 (3 mg/kg) or intra-accumbal DMPX (5 nmol) administration completely abolished reinstatement of heroin-seeking behavior induced by unconditional stimuli (heroin, 0.25 mg/kg). In subsequent studies, using mice with different genotypes, no differences were observed in the cue-induced morphine-seeking behavior after 3 weeks of drug abstinence in the home cage between A_2A_R KO and wild-type animals [101].

The selective A_2A_R antagonist, MSX-3 (1 or 3 mg/kg), as opposed to the effect on heroin-seeking behavior in rats, neither promoted the reinstatement of extinguished THC-seeking behavior, nor altered the reinstatement of drug-seeking behavior induced by priming doses of THC (10 or 40 μg/kg; i.v.) in squirrel monkeys [96].

**Figure 2:**
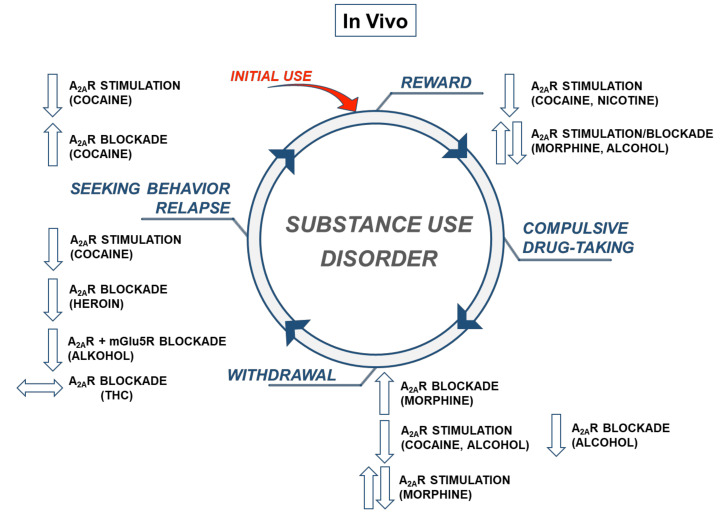
The diagram showing in vivobehavioral effects of stimulation or blockade of the adenosine (A)2A receptors in the cycle of substance use disorders. Up and down arrows indicate an increase or decrease in behavioral responses, respectively. Double-sided horizontal arrows indicate mixed effects on behavioral responses. mGlu5—metabotropic glutamatergic receptors type 5; THC—tetrahydrocannabinol.

In the case of alcohol, there was no direct evidence of A_2A_Rs in ethanol-seeking behavior. However, co-administration of a sub-threshold doses of the A_2A_R antagonist SCH 58261 (0.5 mg/kg) and glutamate mGlu5 receptor antagonist 3-[(2-methyl-1,3-thiazol-4-yl)ethynyl]-pyridine (MTEP; 0.25 mg/kg) effectively blocked conditioned reinstatement of alcohol-seeking in alcohol-preferring rats extinguished from ethanol (10% *v/v*) self-administration [106] (Figure 2).

### 3.5. Withdrawal

There are several preclinical reports on the role of A_2A_Rs in mediating drugs of abuse withdrawal syndromes. For example, O’Neill et al. [115] observed that in rats self-administering cocaine (0.5 mg/kg) under an FR1 schedule of reinforcement, the A_2A_R agonist CGS 21680 (0.03–0.1 mg/kg) reduced the extinction response during the few first extinction sessions, while the A_2A_R antagonist SCH 442416 (0.3–3.0 mg/kg) was inactive during this behavioral paradigm. In turn, a study using a brain stimulation reward task showed that the A_2A_R antagonist DMPX (3–10 mg/kg) reversed the threshold elevation produced by cocaine withdrawal in rats [117].

The initial evidence for the involvement of A_2A_Rs in opioid withdrawal symptoms was inconclusive. Namely, the A_2A_R agonist 2-phenylaminoadenosine (CV-1808; 30 or 100 μg/kg) attenuated naloxone-precipitated withdrawal sings (i.e., teeth chattering or diarrhea) in morphine-dependent rats, but this effect was not dose-dependent [118]. Subsequent studies confirmed the inhibitory effects of the A_2A_R stimulation on some naloxone-precipitated withdrawal symptoms in morphine-dependent rodents. For example, CGS 21680 (0.01 mg/kg) significantly inhibited teeth chattering and forepaw treads in mice [119], and the same agonist (0.03–0.3 mg/kg) decreased in the incidence of body shakes, teeth chattering, and paw shakes in female rats [120]. In turn, a pharmacological blockade of A_2A_Rs with DMPX (1 or 30 mg/kg) increased the incidence of body shakes during morphine withdrawal [119,120].

The inhibitory contribution of A_2A_Rs in the expression of the physical dependence on opioids was further confirmed by studies using the A_2A_R KO mice. Significant increases in naloxone-induced morphine withdrawal somatic signs (the number of jumps, paw tremors, and writhes) in A_2A_R KO compared to wild-type mice were observed [121]. Bilbao and colleagues [122] demonstrated that, while A_2A_R KO mice were characterized by significant increase of diarrhea, sniffing, and the global withdrawal score, the manifestations of naloxone-precipitated morphine withdrawal syndrome were not modified in CB_1_/A_2A_R double KO mice, which may suggest opposite roles of these receptors in the morphine dependence [123]. Equally interesting, a lack of A_2A_Rs abolished quasi-morphine withdrawal syndrome induced by the co-administration of caffeine (an A_1_ and A_2A_ receptors antagonist) and naloxone in opiate-naive mice [122].

Little is known about the role of A_2A_Rs in the control of nicotine, CB, and alcohol withdrawal signs. A separate report indicated that the deletion of A_2A_Rs did not affect the development of the somatic signs of withdrawal induced by the nicotinic receptor antagonist mecamylamine to mice receiving repeated nicotine injections (10 or 25 mg/kg/day for 6 days) by using minipumps [86], suggesting no participation of A_2A_Rs in nicotine withdrawal.

Few reports described A_2A_Rs engagement in THC or alcohol withdrawal symptoms. Soria et al. [82] observed that the A_2A_R deletion significantly attenuated somatic manifestations (global withdrawal score and paw tremors) of CB_1_ receptor antagonist *N*-(piperidin-1-yl)-5-(4-chlorophenyl)-1-(2,4-dichlorophenyl)-4-methyl-1H-pyrazole-3-carboxamide hydrochloride (SR141716A)-precipitated THC withdrawal in mice receiving chronic treatment with THC (20 mg/kg). In turn, alcohol studies found that the A_2A_R agonist CGS 21680 (0.3 mg/kg) reduced the withdrawal score in mice who chronically (14 days) consumed a liquid diet containing ethanol (6.7%, *v/v*) [124], while another agonist (DPMA, 0.1–5 m/kg) did not alter the anxiety-like behavior during ethanol hangovers in mice evaluated in the elevated plus maze test [125].

On the other hand, observations by El Yacoubi and colleagues [126] indicated that a blockade of A_2A_Rs with ZM241385 (20 mg/kg) during the last 5-days of the ethanol intake attenuated ethanol withdrawal-induced seizures in wild-type mice. The handling-induced convulsions score during ethanol withdrawal was lowered in the A_2A_R KO mice when compared with wild-type controls, pointing to A_2A_Rs as a potential target for the treatment of symptoms of alcohol withdrawal.

## 4. A_2A_R and Behavioral Actions to Cocaine and Other Drugs of Abuse—Ex Vivo Evidence

Several preclinical reports using different techniques (i.e., biochemical binding, autoradiography, immunoblotting,and PLA) demonstrated, in theex vivoresearch, changes of the A_2A_R expression in brain areas relevant to repeated exposure to cocaine and other drugs of abuse.

The first quantitative autoradiographic mapping on cocaine showed no difference in the density of A_2A_Rs in the accumbal (core or shell) subregions, dorsal striatum, globus pallidus, and olfactory tubercle in Fischer rats after 14-daily escalating dose "binge" cocaine administration paradigm and a 14-day withdrawal [127]. In 2007, Marcellino and colleagues reported using the A_2A_R antagonist radioligand [^3^H]ZM241385 or the immunoblots that intravenous cocaine self-administration with a discrete trials schedule (4 trials/h) by 10 days evoked an up-regulation in the nucleus accumbens and a down-regulation in the posterior dorsal striatum of functional A_2A_Rs while these changes disappeared to baseline levels after 7 days of drug withdrawal in male Sprague Dawley rats [128]. Another report coming from our laboratory demonstrated that 16 days with i.v. cocaine given to yoked Wistar rats, serving as a control to self-administering cocaine, evoked a significant increase in the A_2A_R density, but not affinity, in the ventral striatum, while the same amount of cocaine self-administered by rats failed.

This increase in the A_2A_R density was still observed following extinction training (10 days) in the yoked group but also appeared in animals previously self-administered cocaine. In contrast to the ventral striatum, no changes were found in the A_2A_R B_max_ values, in the dorsal striatum in both groups of rats with either an active or passive history of cocaine administration and after 10-day extinction training [129]. The increased A_2A_R expression was also reported in the hypothalamus of male Sprague–Dawley rats subjected to 8 and 14 days of cocaine withdrawal following 7-daily s.c. cocaine injections [130].

Recently, we showed that a microinjection of synthetic peptide TM5, a part of the A_2A_R-D_2_ receptor interface, into the nucleus accumbens during cocaine self-administration, blocked in vivo the inhibitory action of the A_2A_R agonist CGS 21680 on cocaine self-administration, and ex vivoreduced the number of A_2A_-D_2_ heteroreceptor complexes within the nucleus accumbens, but not in the dorsal striatum [94]. Using the same PLA method, another transmembrane peptide, TM2, which did not interfere with the formation of the A_2A_-D_2_ heteroreceptor complex, failed to alter CGS 21680-induced inhibition of a cocaine reward or the number of PLA blots identifying A_2A_-D_2_ heteroreceptor complexes in the nucleus accumbens, but it reduced the number of A_2A_-A_2A_homoreceptor complexes in this brain structure in Sprague Dawley rats [93].

The separate report on methamphetamine indicated that experimenter-delivered drugs produced no changes in the A_2A_Rs expression in the striatum, while methamphetamine self-administration for 14 days decreased A_2A_Rs in the accumbens shell and increased them in the amygdala in Sprague Dawley rats [66].

In mice, opposing functional interactions between A_2A_Rs and CB_1_receptors in the hippocampus, as well as the Δ^9^-THC-induced cognitive impairment being blocked by an A_2A_R antagonist, were demonstrated [131]. Such interactions might also occur in the microglia [132]; however, this must be confirmed in further studies.

The opposite findings of the involvement of morphine on the expression of striatal A_2A_Rs indicated that 72-h s.c. delivery of drugs by implanted pump did not change the expression in mice [133], while chronic administration of morphine resulted in a decreased density and affinity of the receptors in rats [134].

Acute or prolonged ethanol exposure and short- or long-term drug withdrawal did not change the A_2A_R numbers and function values in the striatum in mice and rats [124,135,136,137,138,139,140]. Further, long-term voluntary alcohol drinking did not alter gene expression of the accumbal or striatal A_2A_Rs, but significantly increased the number of PLA blots identifying A_2A_-D_2_ heteroreceptor complexes in the accumbal shell and the dorsal striatum in Wistar rats [141].

Adolescent long-term caffeine consumption in adulthood produced decreases in A_2A_R expression with simultaneous increases in D_2_ receptor and protein for DAT and DARPP-32 in the rat nucleus accumbens [142].

To summarize, repeated administration of drugs of abuse evoked changes in the A_2A_R expression seen mainly in the nucleus accumbens and dorsal striatum in rodents and the decreases in these receptors were linked with the local up-regulation of the D_2_receptor or the new A_2A_-D_2_heteroreceptor formation.

## 5. Neurochemical Correlates to Behavioral Findings in Rat Models of Substance Use Disorder. A Focus on Cocaine

The early work of Filip and colleagues [68] indicated that the stimulation of A_2A_Rs can produce inhibitory effects over acute cocaine-induced increases in locomotion and over repeated cocaine-evoked expression of locomotor sensitization in habituated rats. In this paper, the neurochemical mechanism proposed was related to the existence of antagonistic intramembrane A_2A_R-D_2_ receptor interactions in the brain [143] through the formation of A_2A_R-D_2_ receptor heteromeric complexes [30]. The interface between A_2A_R-D_2_ receptor was shown to involve the *C*-terminal tail and the transmembrane helices [144]. More recently, based on the acute effects of cocaine in the nanomolar range (1–100 nM) in the D_2_ receptor-Sigma1 receptor complexes, putative A_2A_-D_2_ receptor-Sigma1 heteroreceptor complexes appeared to have relevance for the treatment of cocaine use disorder [145].

### 5.1. New Mechanisms of Acute Cocaine

As shown in the in vitro studies, cocaine in the nanomolar range enhanced the function of the D_2_ receptor [146,147], related to the existence of D_2_receptor-Sigma1 receptor heterocomplexes and through (i) enhancement of the G_i/o_-mediated signaling of D_2_ receptor protomers via cocaine binding to the Sigma1 receptor and (ii) counteraction of the D_2_receptor protomer internalization HEK293 cells [145]. In the absence of a dominance of A_2A_R-D_2_ receptor-Sigma1 receptor complexes, the action of cocaine likely switches from inhibition to enhancement of D_2_ receptor protomer recognition and signaling.

So far only the dorsal striatum has been studiedin vivo after acute treatment with cocaine. Our present research demonstrated that, in rats, cocaine at a low dose (1 mg/kg)—without increasing the extracellular DA levels—selectively affected the Sigma1 receptors [148]. Further, in the brain tissue of such animals, the A_2A_R agonist CGS 21680 (100 nM) produced significantly larger decreases in the affinity of the high affinity state versus those observed for the saline-treated rats. In the ex vivo studies, cocaine evoked significant reductions of the low and high affinity of D_2_ receptors. Such effects were missing in the control rats. The cocaine induced increases in the antagonistic A_2A_R-D_2_ receptor in the dorsal striatum werelikely due to cocaine-induced increases in the formation of A_2A_R-D_2_ receptor-Sigma1 receptor higher order complexes, in which cocaine can bind to the Sigma1 receptor. The resulting allosteric changes in the D_2_ receptor, through the receptor-protein interaction, may strongly enhance the antagonistic allosteric A_2A_R-D_2_ receptor interactions [148]. Thus, at least in the dorsal striatum acute cocaine in low doses in vivo, not increasing extracellular DA levels, markedly increased the allosteric inhibition of D_2_ receptor recognition.

### 5.2. Cocaine Self-Administration

In rats, cocaine self-administration induced no changes in the A_2A_R density while a 10-day abstinence with extinction training increased the density of A_2A_Rs in the ventral striatum [129]. These observations may be interpreted to reflect a significant and stronger increase of the A_2A_-D_2_ heteroreceptor complex in the ventral striatum in extinction vs. the maintenance of cocaine self-administration. In contrast, in the “yoked” cocaine controls, which received cocaine passively, an increase in the A_2A_R density was developed also in the maintenance phase of cocaine self-administration [129]. This finding may reflect an increase in the antagonistic A_2A_R-D_2_ receptor interactions linked with reduced motivation.

The changes in the A_2A_R density in the ventral striatum appeared to be associated with changes in the overflow of DA, GABA, and glutamate transmitters in cocaine self-administration and its extinction [149]. In fact, during the maintenance of cocaine self-administration, the DA and GABA changes in the accumbal and ventral pallidal overflows may be positively correlated to the motivational features of cocaine intake. On day 10 of extinction (cocaine-free period) reduced basal glutamate extracellular levels were found in the nucleus accumbens [149]. Thus, the panorama of transmitter changes appeared to be different in cocaine self-administration vs. day 10 of extinction. They can in part be related to differential integration of the A_2A_R-D_2_ receptor heterocomplexes in these two states through different types of allosteric receptor–receptor interactions.

Using pharmacological analysis (see above), research demonstrated that A_2A_R antagonists lacked effects on cocaine self-administration while the A_2A_R agonist also gave systemically reduced cocaine rewards and motivation [91]. Thus, there was not an endogenous tone of adenosine activating A_2A_Rs during cocaine self-administration but upon local steady-state infusion of the A_2A_R agonist CGS 21680 an increase in active lever pressing was found instead. These behavioral changes were associated with increased extracellular GABA and reduced DA levels in the nucleus accumbens. Although unexpected, the neurochemical and behavioral data can be explained by the existence of several networks. In these networks, A_2A_Rs control over the cocaine reward and the transmitter events were proposed to be caused by the A_2A_R agonist-induced activation of the post-junctional A_2A_R protomer inhibiting the D_2_ receptor protomer inhibitory signaling in the A_2A_R-D_2_ receptor complex in the ventral striato–pallidal GABA antireward neurons.

In this way, the reduction of D_2_ receptor inhibition of the above-mentioned GABA neurons can explain the increase of extracellular GABA levels observed in the nucleus accumbens[91]. As a consequence, GABA volume transmission can be enhanced, followed by the activation of the GABA receptors, by which, enhanced inhibition of DA release can develop and reach the adjacent DA terminals. Such a mechanism can mediate the reduced extracellular DA levels observed in the nucleus accumbens [91].

In 2016, the first evidence supporting the above hypothesis was obtained using CGS 21680 ex vivo, which significantly reduced the affinity of the D_2_-like receptors in the ventral striatum in self-administering cocaine rats vs. yoked cocaine controls [150]. An inhibitory feedback developed in cocaine self-administration through enhancing the antagonistic allosteric receptor–receptor interactions in the A_2A_R-D_2_receptorheteroreceptor complexes in the ventral striatum. In contrast to the nucleus accumbens, the inhibitory interaction in the A_2A_R-D_2_ receptor complexes in the dorsal striatum was blocked.

The enhancement of D_2_ receptor protomer signaling in the latter brain region may contribute to locomotor sensitization and habit forming learning. Our results demonstrated not only the differential modulation of the A_2A_R-D_2_ receptor heterocomplexes in the ventral vs. the dorsal striatum [148], but suggested that functional plasticity in striatal networks in cocaine addiction could clear-cut increase the antagonistic allosteric A_2A_R-D_2_ receptor interactions in the ventral striatum [151]. Intra-accumbalmicroinjections of CGS 21680 further emphasized the nucleus accumbens as a target for the inhibitory actions of A_2A_R agonists on cocaine self-administration [92]. Microinjections of the A_2A_R agonist into the prefrontal cortex lacked effects on cocaine rewards in self-administration.

The continued work on the neurochemistry of cocaine self-administration demonstrated significant increases in A_2A_R-D_2_ receptor and D_2_ receptor-Sigma1 receptor heterocomplexes in the shell region of the nucleus accumbens [152]. The increase in the D_2_ receptor-Sigma1 receptor complexes in this region may reflect the enhanced formation of A_2A_R-D_2_ receptor-Sigma1 receptor heterocomplexes that, in addition, can produce an increased strength of the antagonistic allosteric A_2A_R-D_2_ receptor interactions [150]. The work of Romieu et al. [153] demonstrated that cocaine could increase the density of Sigma1 receptors in the nucleus accumbens. Cocaine can also bind to the Sigma1 receptor and recruit it into the plasma membrane where the Sigma1 receptor can bind to the D_2_ receptor, increasing the number of D_2_ receptor-Sigma1 receptor complexes in the surface membrane [154,155].

The work on the role of the Sigma1 receptor in cocaine self-administration was resumed using a new pharmacological tool, the monoamine stabilizer OSU-6162, which, in low doses, is a selective Sigma1 receptor ligand with a nanomolar affinity for the receptor [156]. However, in the low dose of 5 mg/kg OSU-6162—when pretreated before cocaine—did not significantly reduce the active lever pressing and the number of cocaine infusions [157]. Only a trend for a reduction of cocaine self-administration was noted after daily injections of OSU-6162 for three days. As shown with competition binding experiments, three days of treatment with OSU-6162 did produce a substantial increase in the density of the D_2_ receptor-Sigma1 receptor complexes in the nucleus shell upon cocaine self-administration vs. the increase found upon treatment with the same OSU-6162 dose in yoked saline animals using ex vivo PLA.

In the same experiment, treatment with OSU-6162 in rats self-administering cocaine evoked significant increases of the A_2A_R-D_2_ receptor heterocomplexes in nucleus accumbens shell vs. the increases obtained for these complexes in the yoked saline animals. The failure to see a significant inhibition of cocaine self-administration with OSU-6162 alone may be the lack of a simultaneous A_2A_R stimulation of the A_2A_R protomer in this experiment. Furthermore, in the D_2_ receptor-Sigma1 receptor complex acute cocaine could enhance D_2_ receptor recognition and signaling with a parallel blockade of the D_2_ receptor internalization [145]. In fact, treatment with the A_2A_R agonist ex vivoin combined treatment within vivoOSU-6162 during cocaine self-administration produced a highly significant reduction of the affinity of the high affinity component of the D_2_ receptor vs. the reduction of affinity of this state found in yoked saline rats not receiving OSU-6162 [157].

### 5.3. Cocaine-Seeking

Reinstatement of cocaine seeking was substantially reduced by A_2A_R agonist treatment with enhanced potency to counteract a D_2_ receptor agonist- or cue-induced cocaine seeking. In contrast, the A_2A_R antagonists KW 6002 and SCH 58261 alone were able to evoke cocaine reinstatement. Preliminary data indicated that the antagonistic allosteric A_2A_R–D_2_ receptor was absent in cocaine reinstatement. Instead, 10-day cocaine abstinence with extinction training was characterized by a strong antagonistic A_2A_R–D_2_ receptor interaction as seen from a clear-cut A_2A_R agonist induced reduction of affinity in the D_2_ receptor high affinity state as studiedex vivo in the ventral striatum. Local microinjections of CGS 21680 into the nucleus accumbens, but not into the intra-prefrontal cortex, strongly reduced cocaine reinstatement, which means that the A_2A_R stimulation controls cocaine seeking and that such interactions depend on the nucleus accumbens [92].

### 5.4. The Role of the A_2A_R-D_2_Receptor Heterocomplexes in Cocaine Use and Addiction

We showed that the synthetic TM5 peptide was part of the interface of the A_2A_R-D_2_ receptor heterodimer in HEK293 cells as it blocked the appearance of the BRET signal and disrupted the rat A_2A_R-D_2_ receptor complex [144,145]. In the in vivo study, the rat synthetic TM5 peptide caused a full blockade of the inhibitory actions of the A_2A_R agonist on cocaine self-administration following its microinjection into the nucleus accumbens, while ex vivo it produced a disappearance of the A_2A_R-D_2_ receptor complex as well as its allosteric receptor–receptor interactions [158]. Taken together, these results gave evidence that the anti-cocaine actions of A_2A_R agonists were mediated through an A_2A_R protomeractivation of an allosteric brake on the D_2_ receptor protomer recognition and signaling in A_2A_R-D_2_ receptor heterocomplexes. The brake appears to be markedly enhanced by activation of the Sigma1 receptor protomer in this receptor complex [158].

At the same time, when the rat synthetic TM5 peptide blocked the inhibitory action of CGS 21680 on the cocaine reward, the rat synthetic TM2 peptide (which neither belongs to the receptor interface of the A_2A_R-D_2_ receptor heterodimer [144,159] nor participates in the interface of the A_2A_R-A_2A_R homodimer [93]) did not block the formation of the A_2A_R-D_2_ receptor heterodimer as determined with a BRET assay and did not interfere with the effects of cocaine in self-administration procedures. We reported that the nucleus accumbens microinjection with TM2 peptide reduced the formation of the A_2A_R homodimer, however, this had no consequences for the inhibitory actions of the A_2A_R agonist on cocaine self-administration [93]. Thus, disruption of the A_2A_R-A_2A_R homomer caused no effects on rat cocaine self-administration and the A_2A_R homomeric complex did not appear to have a critical role in cocaine use and addiction.

A transgenic approach based on using rats with the overexpression of A_2A_R under the regulation of the neuronal specific enolase promotor (NSEA2A) indicated an enhanced number of such receptor complexes with possible increased stoichiometry mainly in the prefrontal cortex, the hippocampal formation, the striatum, and the cerebellum [160]. Such enhancement could lead to increased A_2A_R signaling, which mediates the substantial inhibitory effects on nicotine behavioral readouts, such as sensitization of locomotion or conditioned locomotor activity (see above). These observations indicated that enhanced A_2A_R signaling in the above brain areas could also counteract nicotine use.

## 6. Final Conclusions

The latest preclinical findings indicated a role for A_2A_Rs in several models to assess drug reward, withdrawal, or seeking behavior. Several rodent research models clearly demonstrated that the stimulation of A_2A_Rs was specific to cocaine for the attenuation of drug-induced rewarding effects, withdrawal syndrome, and relapses. Unlike cocaine, no similar effects were seen for other drugs of abuse regarding reward or seeking behaviors. Concerning drug withdrawal,studies reported that the A_2A_R stimulation was effective for reducing opioid or alcohol withdrawal symptoms. The research information on the interaction A_2A_R-nicotine or A_2A_R-cannabinoids is still limited and does not allow us to make conclusions regarding the receptors control over the behavioral or neurochemical outcomes of the above drugs of abuse.

The interaction between A_2A_Rs and cocaine seen at the behavioral level was associated with the antagonistic interplay within A_2A_R-D_2_ receptor complexes localized in the central striato–pallidal GABA neurons as shown ex vivoat both the molecular and behavioral levels. This observation should prompt further preclinical research on the study of antagonistic interactions of the A_2A_R-D_2_ receptors for investigations on other drugs of abuse as well asresearch in the search for safe and effective anti-cocaine A_2A_R agonists and/or heterobivalent ligands targeted for A_2A_R-D_2_receptors.
